# Knowledge, perceptions, attitudes, and clinical experiences on molar incisor hypomineralization among Syrian pediatric dentists and general dental practitioners: a cross-sectional study

**DOI:** 10.1186/s12903-022-02620-5

**Published:** 2022-12-01

**Authors:** Mawia Karkoutly, Blend Hamza, Sami Al Batal, Amat Al Barazi, Nada Bshara

**Affiliations:** 1 Pediatric Dentistry Department, Dental College, Damascus University, Damascus, Syrian Arab Republic; 2 Clinic of Orthodontics and Pediatric Dentistry, Center of Dental Medicine, University of Zurich, Zurich, Switzerland; 3 Dental College, Damascus University, Damascus, Syrian Arab Republic

**Keywords:** Molar incisor hypomineralization, Knowledge, Perceptions, Attitudes, Clinical experiences, Oral health problem, General dental practitioners, Pediatric dentists, Questionnaire

## Abstract

**Background:**

Molar incisor hypomineralization (MIH) is a widespread oral health problem. Dentists encounter several challenges regarding MIH management worldwide. The aim of this study was to evaluate and compare the knowledge, perceptions, attitudes, and clinical experiences on MIH among general dental practitioners and pediatric dentists in Syria.

**Methods:**

All general dental practitioners and pediatric dentists belonging to the Syrian Dental Syndicate of Damascus were invited to complete a cross-sectional structured questionnaire (n = 1936). The questionnaire consisted of four sections and required responses regarding demographic data, knowledge, perceptions, attitudes, and experiences on MIH. Data were analyzed with Pearson’s chi-square test and multivariate regression models using SPSS Ver. 23.0.

**Results:**

The overall response rate was 36.31% (703/1936). Pediatric dentists were significantly more familiar with MIH (*p* < 0.001) and more confident when diagnosing it (*p* < 0.001). Most participants (43.95%) perceived an increase in MIH prevalence in Syria. Stainless steel crowns were the most favorable restorative material for molars with post-eruptive breakdown (51.38%). As for molars and incisors with opacities, composite resin was preferred with (41.82%), and (67.51%) respectively. General dental practitioners requested further training regarding MIH treatment (*p* < 0.001).

**Conclusions:**

Pediatric dentists were equipped with further knowledge regarding MIH, and were more confident when diagnosing it. There is a need for additional training and education for general dental practitioners. Most respondents perceived an increase in the prevalence of MIH. There is a dearth of data regarding MIH prevalence in Syria. The materials of choice for restoring teeth with MIH were stainless steel crowns and composite resin.

## Background

Molar incisor hypomineralization (MIH) was first described by Weerheijm et al. [1] in 2001 and refers to qualitative developmental enamel defects, which affect one or more first permanent molars and less frequently associated with the involvement of upper permanent incisors. It clinically presents as white–creamy or yellow–brown demarcated opacities, and it is combined with structural loss resulting in post-eruptive enamel breakdown (PEB) in severely affected enamel [2]. In 2018, the global prevalence of MIH was 14.2%. Furthermore, the estimated prevalence of MIH amongst children aged 10 years or younger was 15.1%, and the older had a lower prevalence (12.1%) [3]. In Syria, there is a lack of data regarding MIH prevalence among Syrian children. The definitive etiological factors of MIH are still to be determined. However, genetic factors, acute or chronic medical conditions, medications, childhood illness, and birth complications are the most putative factors related to MIH [2, 4, 5]. MIH clinical management poses a serious challenge for both dentists and patients due to determining the suitable preparation margin, selecting the optimal restorative material, esthetic issues, teeth hypersensitivity, achieving adequate pain control, and managing dental anxiety [6, 7]. According to a clinical study in three UK dental hospitals, MIH was the second cause of the first permanent molar extraction following dental caries [8]. Hence, MIH can negatively affect children’s quality of life and cause impaired oral health [9]. The aforementioned facts highlight the essential role of physicians in MIH appropriate management and resolving patients’ anxiety. However, despite the high global prevalence, poor oral health-related quality of life (OHRQoL), and challenging clinical management related to MIH, no study has ever evaluated the perception of Syrian clinicians of this alarming problem. Thus, the aim of this study was to compare and evaluate the knowledge, perceptions, attitudes, and clinical experiences of pediatric dentists and general dental practitioners regarding MIH in Syria using a questionnaire. Such questionnaires point out if there is a knowledge gap among dentists regarding MIH and shed light on the necessity of providing training programs concerning MIH clinical management.

## Material and methods

### Participants and procedures

Ethical approval was obtained from the institutional review board of Damascus University (N 223/2022) prior to data collection, and the study was performed in accordance with the Declaration of Helsinki. Participants were pediatric dentists (PDs) and general dental practitioners (GDPs) who were members of the Syrian Dental Syndicate of Damascus. Google Forms software survey was used to create an online Arabic questionnaire. A questionnaire was designed based on existing validated questionnaires [10–13]. The questionnaire was first piloted by a group of PDs and GDPs to ensure that the questions were easy to understand and took no longer than 5 min to complete. “Not sure” choice was added for questions regarding restorative material selection. In addition, “fluoride” and “sealant” options were added for the second question in the clinical problems section.

An Arabic version of the questionnaire was distributed to Damascus Dental Syndicate members (n = 1936) via email in March 2022. The email declared that participation was anonymous and optional, and the researchers had no access to the participants’ personal data. It was also distributed via social networks (Facebook, Twitter, Whatsapp, etc.). The questionnaire was online for 2 months. The inclusion criteria for questionnaire participants were: (1) members of Damascus Dental Syndicate, (2) GDPs, (3) PDs.

## Questionnaire instruments

**Fig. 1:**
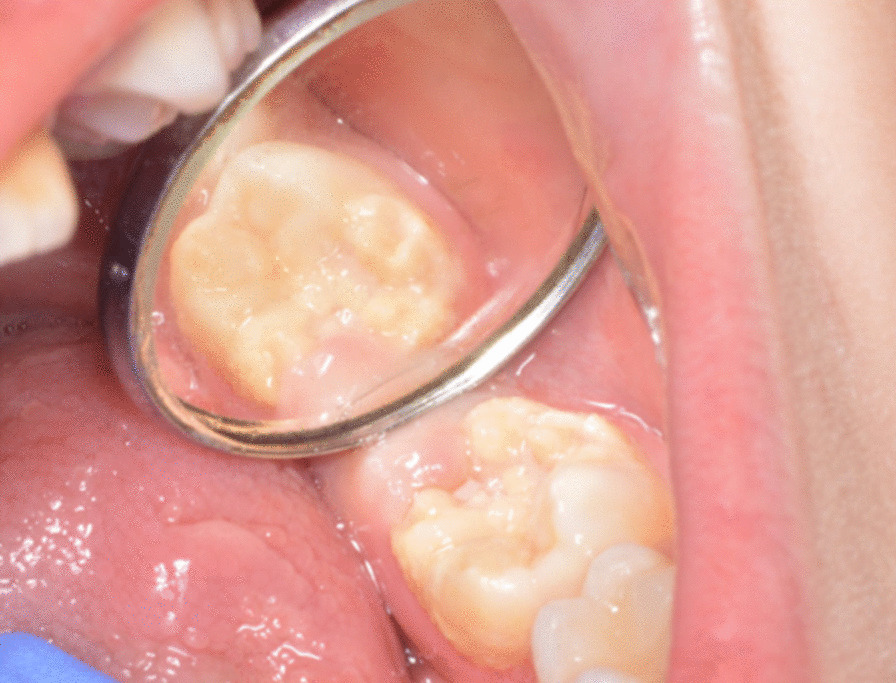
A 7-year-old child was referred to your clinic with semi erupted MIH-affected tooth with enamel post eruptive breakdown (PEB) and sensitivity. What is the most optimal treatment option in your opinion? Image and question. Serna Munoz et al. [10]

**Fig. 2:**
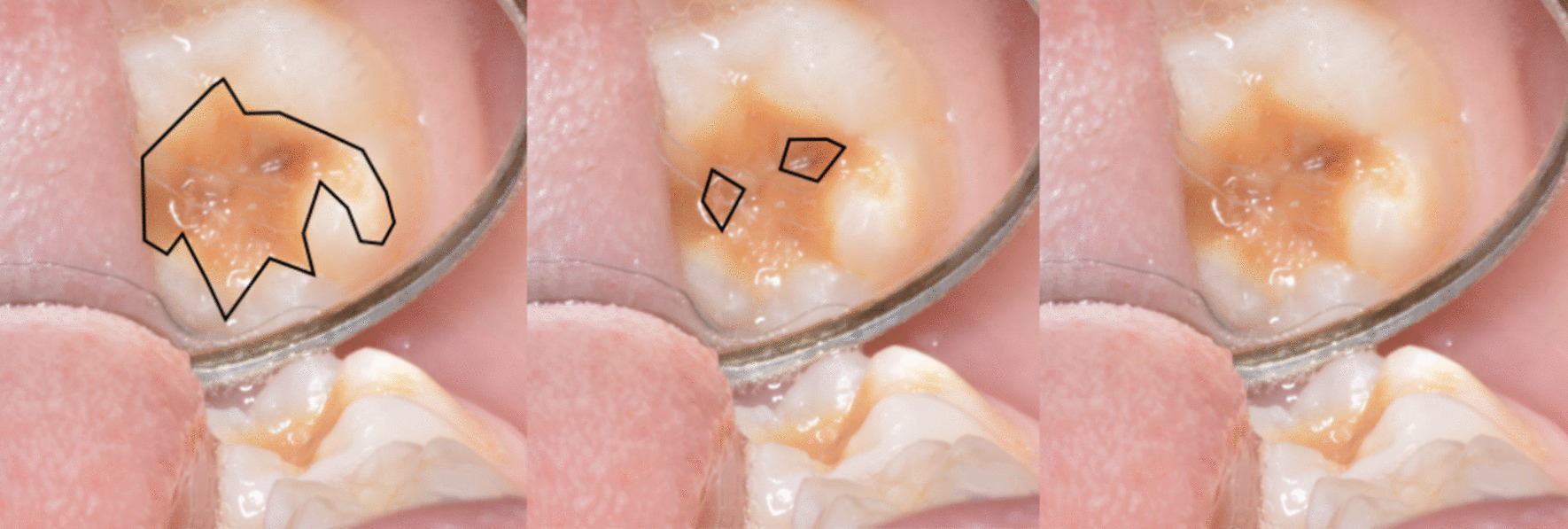
Where would you place preparation margin for a molar with delimited brown opacity without post-eruptive enamel fracture, and which restorative material would you prefer? Image and question. Serna Munoz et al. [10]

The questionnaire consisted of four sections. The first section included a brief definition of MIH associated with clinical photographs and a reminder of the anonymous and voluntary participation. The second section collected sociodemographic data including sex, age, years of practice, and work sector. The third section addressed participants’ knowledge about MIH, differential diagnosis, and possible etiological factors related to MIH. Dentists were also asked if they felt confident when diagnosing MIH. Furthermore, it addressed dentists’ perception of MIH prevalence, frequency of MIH occurrence in permanent teeth, and challenges encountered by both children and their parents regarding dental visits. The section also covered participants’ practices and clinical experiences on MIH including favorable restorative material choices and influencing factors, most noticed clinical appearance, referral decisions to a specialist, and difficulties concerning MIH management. Participants were also asked if they were using a specific index for diagnosis and treatment. In addition, it covered participants’ attitudes toward continuous education regarding MIH. The last section included two clinical cases associated with photographs as used in a similar study [10]. Dentists were asked for the best treatment option for both clinical cases (Figs. 1, 2).

### Statistical analysis

Data were entered into an excel spreadsheet (Microsoft Excel, Microsoft Corp, WA, USA) by Google Forms. Statistical analysis was performed using IBM SPSS software v. 23 (IBM Corp., Armonk, USA). Descriptive analysis (simple frequency distribution, and percentage) was determined. Significant differences between the two study groups (GDPs, and PDs) were checked using Pearson’s chi-square test. The level of statistical significance was set at 0.05 (*p* < 0.05). Multivariate regression models were used to assess the relationship between independent variables (years of practice, and the frequency of encountering MIH patients) and restorative material selection as a parameter. For the multivariate model, the statistical significance level was set as 0.25 as the 0.05 level of significance can fail in identifying important variables [14].

## Results

Of the 1936 dentists invited to complete the online questionnaire, 705 responded. Two questionnaires with missing answers were excluded, the overall response rate was 36.31% (703/1936). A response rate of 34.42% (578/1679) was achieved for the GDPs and 48.63% (125/257) for the PDs.

### Demographic data of the participants

Table 1 shows the demographic characteristics. Of the participants, 82.21% were GDPs, 17.78% were PDs, 54.20% were male, and 45.80% were female. Most GDPs (36.51%), and PDs (43.20%) were 26–30 year old. Similarly, most GDPs (67.65%), and PDs (44.00%) had fewer than 5 years of practice. More than half of the GDPs (59.86%), and PDs (45.60%) worked in the private sector, while the remaining participants were distributed across public (GDPs: 15.57% vs. PDs: 25.60%) and combined sectors (GDPs: 24.57% vs. PDs: 28.80%).

**Table 1:** Demographic data of study participants

Characteristics	Total, n (%)	GDPs, n (%)	PDs, n (%)
Sex	703 (100)	578 (100)	125 (100)
Male	381 (54.20)	321 (55.54)	60 (48.00)
Female	322 (45.80)	257 (44.46)	65 (52.00)
Age	703 (100)	578 (100)	125 (100)
≤ 25	234 (33.39)	204 (35.29)	30 (24.00)
26–30	265 (37.70)	211 (36.51)	54 (43.20)
31–40	103 (14.65)	83 (14.36)	20 (16.00)
41–50		36 (6.23)	5 (4.00)
≥ 51	60 (8.53)	44 (7.61)	16 (12.80)
Years of practice	703 (100)	578 (100)	125 (100)
≤ 5	446 (63.44)	391 (67.65)	55 (44.00)
6–10	111 (15.79)	72 (12.46)	39 (31.20)
11–15	47 (6.69)	42 (7.27)	5 (4.00)
> 15	99 (14.08)	73 (12.63)	26 (20.80)
Work sector	703 (100)	578 (100)	125 (100)
Public sector	122 (17.35)	90 (15.57)	32 (25.60)
Private sector	403 (57.33)	346 (59.86)	57 (45.60)
Combined	178 (25.32)	142 (24.57)	36 (28.80)

GDPs, general dental practitioners; PDs, pediatric dentists

### Knowledge about MIH

Participants’ knowledge regarding MIH is listed in Table 2. PDs were significantly more familiar with MIH (*p* < 0.001), more confident when diagnosing MIH (*p* < 0.001), and implemented clinical criteria to diagnose MIH (*p* < 0.001) compared to GDPs. A good proportion of participants (52.22%) reported that amelogenesis imperfecta was difficult to distinguish from MIH. However, enamel hypoplasia was the only defect that showed a significant difference between the groups (*p* = 0.003). Regarding the etiological factors of MIH, most participants believed that genetic factors (GDPs: 64.90% vs. PDs: 45.53%) and acute medical conditions during pregnancy (GDPs: 48.74% vs. PDs: 54.46%) were involved in the etiology of MIH.

**Table 2:** Participants’ knowledge about MIH

Question	Total, n (%)	GDPs, n (%)	PDs, n (%)	*P* value
1. Are you familiar with MIH?	703 (100)	578 (100)	125 (100)	< 0.001*
Yes	471 (67.00)	359 (62.11)	112 (89.60)	
No	232 (33.00)	219 (37.89)	13 (10.40)	
2. How confident do you feel when diagnosing MIH?	471 (100)	359 (100)	112 (100)	< 0.001*
Not confident	33 (7.01)	28 (7.80)	5 (4.46)	
Slightly confident	190 (40.34)	155 (43.18)	35 (31.25)	
Confident	194 (41.19)	160 (44.57)	34 (30.36)	
Very confident	54 (11.46)	16 (4.46)	38 (33.93)	
3. Do you know if there are clinical criteria to diagnose MIH?	471 (100)	359 (100)	112 (100)	< 0.001*
No	115 (24.42)	109 (30.36)	6 (5.36)	
Yes, but I do not know how to implement them	233 (49.47)	184 (51.25)	49 (43.75)	
Yes, and I know how to implement them	123 (26.11)	66 (18.38)	57 (50.89)	
4. Which malformations do you find particularly difficult to distinguish from MIH?^a^	471 (100)	359 (100)	112 (100)	
Amelogenesis imperfect	246 (52.22)	188 (52.36)	58 (51.78)	0.914
Enamel hypoplasia	187 (39.70)	156 (43.45)	31 (27.67)	0.003*
Dentinogenesis imperfect	109 (23.14)	87 (24.23)	22 (19.64)	0.315
Dental fluorosis	178 (37.79)	144 (40.11)	34 (30.35)	0.063
Dental caries	47 (9.97)	32 (8.91)	15 (13.39)	0.167
Local defects	125 (26.53)	90 (20.06)	35 (31.25)	0.196
5. Which factors do you think are involved in the etiology of MIH?^a^	471 (100)	359 (100)	112 (100)	
Genetic factors	284 (60.29)	233 (64.90)	51 (45.53)	< 0.001*
Acute medical condition that affects the mother during pregnancy	236 (50.10)	175 (48.74)	61 (54.46)	0.291
Acute medical condition that affects the child involved	134 (28.45)	91 (25.34)	43 (38.39)	0.008*
Antibiotics/medications taken by the mother during pregnancy	201 (42.67)	169 (47.07)	32 (28.57)	0.001*
Antibiotics/medications taken by the child involved	93 (19.74)	62 (17.27)	31 (27.67)	0.016*
Chronic medical condition that affects the mother during pregnancy	121 (25.69)	98 (27.29)	23 (20.53)	0.153
Chronic medical condition that the child involved	85 (18.04)	55 (15.32)	30 (26.78)	0.006*
Environmental contaminants	132 (28.02)	100 (27.85)	32 (28.57)	0.883
Fluoride exposure	101 (21.44)	87 (24.23)	14 (12.50)	0.008*
Not sure	36 (7.64)	32 (8.91)	4 (3.57)	0.063

GDPs, general dental practitioners; PDs, pediatric dentists

*p < 0.05 = significant difference; p values written in bold are statistically significant (p < 0.05)

^a^Multiple-choice questions

### Perceptions of MIH

Perceptions of responding participants of MIH are presented in Table 3. 39.83% of GDPs had noticed hypomineralized teeth annually, while most of PDs (36.61%) had made diagnosis of MIH weekly (*p* < 0.001). In addition, 67.97% of GDPs, and 58.93% of PDs reported that < 10% of their patients presented MIH (*p* = 0.001). Yellow–brown demarcated opacities were the most clinical defects noticed by participants (*p* = 0.006), followed by white demarcated opacities and post-eruptive enamel breakdown. In terms of prevalence, 64.29% of PDs claimed that MIH prevalence had been increasing in Syria in recent years, with a significant difference to GDPs (*p* < 0.001). Regarding patients’ quality of life, there were significant differences between the two groups for the problem of pain (*p* = 0.043), appearance (*p* = 0.040), anxiety (*p* = 0.005), and missing school (*p* = 0.023). PDS were more concerned than GDPs about the pain experienced and missing school, while GDPs were more concerned about the anxiety experienced and the appearance of the defect. Furthermore, 22.29% of the participants perceive parents’ anxiety toward dental treatment under general anesthesia.

**Table 3:** Participants’ perception of MIH

Question	Total, n (%)	GPDs, n (%)	PDs, n (%)	*P* value
6. How often do you notice hypomineralised teeth in your practice?	471 (100)	359 (100)	112 (100)	< 0.001*
Weekly	69 (14.65)	28 (7.80)	41 (36.61)	
Monthly	173 (36.73)	139 (38.72)	34 (30.36)	
Annually	162 (34.39)	143 (39.83)	19 (16.96)	
Never	67 (14.23)	49 (13.65)	18 (16.07)	
7. Approximately what percentage of your patients present this malformation?	471 (100)	359 (100)	112 (100)	
0%	48 (10.19)	43 (11.98)	5 (4.46)	0.001*
< 10%	310 (65.82)	244 (67.97)	66 (58.93)	
10–25%	101 (21.44)	66 (18.38)	31.25)	
> 25%	12 (2.55)	6 (1.67)	6 (5.36)	
8. Do you perceive that the prevalence of MIH has increased in recent years?	471 (100)	359 (100)	112 (100)	< 0.001*
Yes	207 (43.95)	135 (37.60)	72 (64.29)	
No	70 (14.86)	50 (13.93)	20 (17.86)	
Not sure	194 (41.19)	174 (48.47)	20 (17.86)	
9. What do you most frequently notice in your practice?^a^	471 (100)	359 (100)	112 (100)	
White demarcated opacities	212 (45.01)	168 (46.79)	44 (39.28)	0.163
Yellow–brown demarcated opacities	306 (64.96)	221 (61.55)	85 (75.89)	0.006*
Post eruptive eruption	156 (33.12)	122 (33.98)	34 (30.35)	0.477
10. How much of a problem to children are	471 (100)	359 (100)	112 (100)	
Pain				
Never/almost never	67 (14.23)	53 (14.76)	14 (12.50)	0.043*
Sometimes	185 (39.28)	146 (40.67)	39 (34.82)	
Often	149 (31.63)	116 (32.31)	33 (29.46)	
Almost always	70 (14.86)	44 (12.26)	26 (23.21)	
Appearance				
Never/almost never	35 (7.43)	25 (6.96)	10 (8.93)	0.040*
Sometimes	146 (31.00)	114 (31.75)	32 (28.57)	
Often	193 (40.98)	137 (38.16)	56 (50.00)	
Almost always	97 (20.59)	83 (23.12)	14 (12.50)	
Anxiety				
Never/almost never	13 (2.76)	7 (1.95)	6 (5.36)	0.005*
Sometimes	84 (17.83)	54 (15.04)	30 (26.79)	
Often	172 (36.52)	138 (38.44)	34 (30.36)	
Almost always	202 (42.89)	160 (44.57)	42 (37.50)	
Numerous visits				0.776
Never/almost never	32 (6.79)	22 (6.13)	10 (8.93)	
Sometimes	148 (31.42)	114 (31.75)	34 (30.36)	
Often	181 (38.43)	138 (38.44)	43 (38.39)	
Almost always	110 (23.35)	85 (23.68)	25 (22.32)	
Missing school				0.023*
Never/almost never	143 (30.36)	108 (30.08)	35 (31.25)	
Sometimes	211 (44.80)	171 (47.63)	40 (35.71)	
Often	72 (15.29)	53 (14.76)	19 (16.96)	
Almost always	45 (9.55)	27 (7.52)	18 (16.07)	
11. How much of a problem to parents are	471 (100)	359 (100)	112 (100)	
Difficulty eating/drinking				
Never/almost never	60 (12.74)	43 (11.98)	17 (15.18)	0.180
Sometimes	210 (44.59)	166 (46.24)	44 (39.29)	
Often	141 (29.94)	110 (30.64)	31 (27.68)	
Almost always	60 (12.74)	40 (11.14)	20 (17.86)	
Getting teased				0.319
Never/almost never	40 (8.49)	26 (7.24)	14 (12.50)	
Sometimes	189 (40.13)	143 (39.83)	46 (41.07)	
Often	182 (38.64)	143 (39.83)	39 (34.82)	
Almost always	60 (12.74)	47 (13.09)	13 (11.61)	
Anxiety				
Never/almost never	30 (6.37)	17 (4.74)	13 (11.61)	0.061
Sometimes	156 (33.12)			
Often	188 (39.92)	149 (41.50)	39 (34.82)	
Almost always	97 (20.59)	75 (20.89)	22 (19.64)	
Time off work				0.916
Never/almost never	72 (15.29)	57 (15.88)	15 (13.39)	
Sometimes	218 (46.28)	165 (45.96)	53 (47.32)	
Often	139 (29.51)	106 (29.53)	33 (29.46)	
Almost always	42 (8.92)	31 (8.64)	11 (9.82)	
Missing school				0.229
Never/almost never	70 (14.86)	50 (13.93)	20 (17.86)	
Sometimes	192 (40.76)	151 (42.06)	41 (36.61)	
Often	150 (31.85)	118 (32.87)	32 (28.57)	
Almost always	59 (12.53)	40 (11.14)	19 (16.96)	
General anesthesia				0.205
Never/almost never	52 (11.04)	36 (10.03)	16 (14.29)	
Sometimes	167 (35.46)	128 (35.65)	39 (34.82)	
Often	147 (31.21)	108 (30.08)	39 (34.82)	
Almost always	105 (22.29)	87 (24.23)	18 (16.07)	

GDPs, general dental practitioners; PDs, pediatric dentists

*p < 0.05 = significant difference; p values written in bold are statistically significant (p < 0.05)

^a^Multiple-choice questions

### Practices and clinical experiences on MIH

Table 4 shows participants’ practices and clinical experiences on MIH. Stainless steel crowns were the material of choice for molars with post-eruptive fractures (GDPs: 51.81% vs PDs: 50.00%). However, there were significant differences to GDPs in the use of flowable composite resin (*p* = 0.001), and Silver diamine fluoride (*p* = 0.006). In contrast, there was a significant difference to PDs in the use of resin modified glass ionomer cement (RMGIC) (*p* = 0.022). As for molars with opacities, composite resin was the material of choice (GDPs: 44.01% vs PDs: 34.82%), with significant differences to GDPs in the use of compomer (*p* < 0.001). Similarly, composite resin was the material of choice for incisors with opacities (GDPs: 67.40% vs PDs: 67.85%). However, there were significant differences to GDPs in the use of flowable composite resin (*p* = 0.024). Durability was the main factor when deciding which material to use among participants (GDPs: 77.43% vs. PDs: 73.21%), with significant differences to PDs in adhesion (*p* < 0.001), and esthetics (*p* = 0.005) factors. In contrast, there was a significant difference to GDPs in experience in choosing restorative materials (*p* = 0.021). The multivariate regression model demonstrated that composite resin, stainless steel crown, and glass ionomer cement (GIC) were significantly preferred by participants with more than 15 years of practice for molars with opacities. Moreover, RMGIC was significantly less preferred by participants who have more than 15 years of practice for molars and incisors with opacities. In addition, composite resin was significantly less preferred by participants with less than 5 years of practice for incisors with opacities. For molars with post-eruptive eruption, years of practice was not a predictor for restorative material selection. However, the frequency of encountering MIH patients was a predictor for the latter parameter and flowable composite resin was significantly the most preferable material for dentists who encounter MIH patients weekly. In addition, they prefered stainless steel crowns and composite resin restorations for molars with post-eruptive breakdown and incisors with opacities. Regarding MIH referral considerations, approximately half of GDPs (53.20%) would refer a child with MIH signs to a dental specialist, while most of PDs preferred treating MIH-affected children themselves (*p* < 0.001). More than half of PDs (58.93%) used a specific diagnosis index, compared to only 8.64% of the GDPs (*p* < 0.001). Regarding clinical management difficulties, more than the third of the participants said that esthetics (GDPs: 33.15% vs. PDs: 41.96%) and long-term success of restoration (GDPs: 38.16% vs. PDs: 42.86%) were often challenging issues (Table 4).

**Table 4:** Participants’ practices and clinical experiences on MIH

Question	Total, n (%)	GDPs, n (%)	PDs, n (%)	*P* value
12. Material of choice for molars with post-eruptive fractures^a^	471 (100)	359 (100)	112 (100)	
Compomer	77 (16.34)	59 (16.43)	18 (16.07)	0.928
Composite resin	159 (33.75)	124 (34.54)	35 (31.25)	0.520
Flowable composite resin	53 (11.25)	31 (8.63)	22 (19.64)	0.001*
Stainless steel crown	242 (51.38)	186 (51.81)	56 (50.00)	0.738
Silver diamine fluoride	35 (7.43)	20 (5.57)	15 (13.39)	0.006*
Cast restoration	45 (9.55)	36 (10.02)	9 (8.03)	0.531
Glass ionomer cement	86 (18.25)	72 (20.05)	14 (12.5)	0.071
Resin modified glass ionomer cement	137 (29.08)	114 (31.75)	23 (20.53)	0.022*
Not sure	36 (7.64)	32 (8.91)	4 (3.57)	0.063
Other	6 (1.27)	5 (1.39)	1 (0.89)	0.680
13. Material of choice for molars with opacities^a^	471 (100)	359 (100)	112 (100)	
Amalgam	58 (12.31)	47 (13.09)	11 (9.82)	0.358
Compomer	73 (15.49)	42 (11.69)	31 (27.67)	< 0.001*
Composite resin	197 (41.82)	158 (44.01)	39 (34.82)	0.085
Flowable composite resin	81 (17.19)	64 (17.82)	17 (15.17)	0.517
Stainless steel crown	88 (18.68)	66 (18.38)	22 (19.64)	0.765
Silver diamine fluoride	42 (8.91)	28 (7.79)	14 (12.50)	0.128
Glass ionomer cement	64 (13.58)	47 (13.09)	17 (15.17)	0.574
Resin modified glass ionomer cement	114 (24.20)	91 (25.34)	23 (20.53)	0.299
Fluoride	72 (15.28)	49 (13.64)	23 (20.53)	0.077
Sealant	55 (11.67)	41 (11.42)	14 (12.50)	0.756
Not sure	39 (8.28)	34 (9.47)	5 (4.46)	0.093
14. Material of choice for incisors with opacities^a^	471 (100)	359 (100)	112 (100)	
Compomer	76 (16.13)	51 (14.20)	25 (22.32)	0.042
Composite resin	318 (67.51)	242 (67.40)	76 (67.85)	0.930
Flowable composite resin	77 (16.34)	51 (14.20)	26 (23.21)	0.024*
Stainless steel crown	20 (4.24)	16 (4.45)	4 (3.57)	0.685
Silver diamine fluoride	31 (6.58)	22 (6.12)	9 (8.03)	0.477
Glass ionomer cement	58 (12.31)	45 (12.53)	13 (11.60)	0.794
Resin modified glass ionomer cement	110 (23.35)	90 (25.06)	20 (17.85)	0.115
Not sure	38 (8.06)	33 (9.19)	5 (4.46)	0.109
Other	16 (3.39)	7 (1.94)	9 (8.03)	0.002*
15. Factors in the choice of material^a^	471 (100)	359 (100)	112 (100)	< 0.001*
Adhesion	272 (57.74)	225 (62.67)	47 (41.96)	
Durability	360 (76.43)	278 (77.43)	82 (73.21)	0.358
Experience	113 (23.99)	77 (21.44)	36 (32.14)	0.021*
Remineralization potential	156 (33.12)	126 (35.09)	30 (26.78)	0.103
Patient/parent preferences	74 (15.71)	51 (14.20)	23 (20.53)	0.108
Sensitivity	156 (33.12)	119 (33.14)	37 (33.03)	0.982
Research findings	68 (14.43)	51 (14.20)	17 (15.17)	0.798
Esthetics	244 (51.80)	199 (55.43)	45 (40.17)	0.005*
Not sure	13 (2.76)	12 (3.34)	1 (0.89)	0.167
16. Would you refer a child who has MIH to a specialist?	471 (100)	359 (100)	112 (100)	< 0.001*
Yes	225 (47.77)	191 (53.20)	34 (30.35)	
No	183 (38.85)	153 (42.61)	30 (26.78)	
I am working as a pediatric dentist	63 (13.37)	15 (4.17)	48 (42.85)	
17. Do you use a specific index for MIH?	471 (100)	359 (100)	112 (100)	
Yes	97 (20.59)	31 (8.64)	66 (58.93)	< 0.001*
No	374 (79.41)	328 (91.36)	46 (41.07)	
18. How much of a challenge have the following issues been to you?	471 (100)	359 (100)	112 (100)	
Diagnosis				
Never/almost never	102 (21.66)	65 (18.11)	37 (33.04)	
Sometimes	273 (57.96)	219 (61.00)	54 (48.21)	< 0.001*
Often	76 (16.14)	65 (18.11)	11 (9.82)	
Almost always	20 (4.25)	10 (2.79)	10 (8.93)	
Esthetics				
Never/almost never	79 (16.77)	64 (17.83)	15 (13.39)	
Sometimes	188 (39.92)	147 (40.95)	41 (36.61)	
Often	166 (35.24)	119 (33.15)	47 (41.96)	0.349
Almost always	38 (8.07)	29 (8.08)	9 (8.04)	
Long-term success of restoration				
Never/almost never	46 (9.77)	35 (9.75)	11 (9.82)	
Sometimes	187 (39.70)	151 (42.06)	36 (32.14)	0.203
Often	185 (39.28)	137 (38.16)	48 (42.86)	
Almost always	53 (11.25)	36 (10.03)	17 (15.18)	
Correct determination of restoration margins				
Never/almost never	81 (17.20)	67 (18.66)	14 (12.50)	
Sometimes	207 (43.95)	165 (45.96)	42 (37.50)	0.044*
Often	137 (29.09)	96 (26.74)	41 (36.61)	
Almost always	46 (9.77)	31 (8.64)	15 (13.39)	
Achieving correct local anesthetic				
Never/almost never	252 (53.50)	216 (60.17)	36 (32.14)	
Sometimes	113 (23.99)	82 (22.84)	31 (27.68)	< 0.001*
Often	74 (15.71)	43 (11.98)	31 (27.68)	
Almost always	32 (6.79)	18 (5.01)	14 (12.50)	
Providing correct restoration				
Never/almost never	120 (25.48)	99 (27.58)	21 (18.75)	
Sometimes	209 (44.37)	165 (45.96)	44 (39.29)	
Often	117 (24.84)	79 (22.01)	38 (33.93)	
Almost always	25 (5.31)	16 (4.46)	9 (8.04)	0.015*

GDPs, general dental practitioners; PDs, pediatric dentists

*p < 0.05 = significant difference; p values written in bold are statistically significant (p < 0.05)

^a^Multiple-choice questions

### Attitudes toward MIH

Table 5 shows dentists’ attitudes toward MIH. The majority of GDPs (89.14%) did not receive any information on MIH, while 62.50% of PDs did (*p* < 0.001). Among those who received information on MIH, continuous education (GDPs: 58.97% vs. PDs: 70.00%) was the main source of knowledge, followed by the internet (GDPs: 58.97% vs. PDs: 42.85%) and journals (GDPs: 48.71% vs. PDs: 48.57%). Almost three-quarters of the participants agreed on the necessity of information in the field of diagnosis (GDPs: 78.55% vs. PDs: 72.32%) and treatment (GDPs: 81.05% vs. PDs: 62.50%) of MIH.

**Table 5:** Participants’ attitude toward MIH

Question	Total, n (%)	GPDs, n (%)	PDs, n (%)	*P* value
22. Do you receive any information on MIH?	471 (100)	359 (100)	112 (100)	< 0.001*
Yes	109 (23.14)	39 (10.86)	70 (62.50)	
No	362 (76.86)	320 (89.14)	42 (37.50)	
23. Where do you obtain the information?^a^	109 (100)	39 (100)	70 (100)	
Journals	53 (48.62)	19 (48.71)	34 (48.57)	0.988
Continuing education	72 (66.05)	23 (58.97)	49 (70.00)	0.244
Brochures	20 (18.34)	3 (7.69)	17 (24.28)	0.032*
Internet	53 (48.62)	23 (58.97)	30 (42.85)	0.107
Books	24 (22.01)	9 (23.07)	15 (21.42)	0.842
Other	3 (2.75)	0 (0.00)	3 (4.28)	0.190
24. Where do you think more information is necessary?^a^	471 (100)	359 (100)	112 (100)	
Etiology	234 (49.68)	183 (50.97)	51 (45.53)	0.315
Diagnosis	363 (77.07)	282 (78.55)	81 (72.32)	0.171
Treatment	361 (76.64)	291 (81.05)	70 (62.5)	< 0.001*
Other	6 (1.27)	4 (1.11)	2 (1.78)	0.580

GDPs, general dental practitioners; PDs, pediatric dentists; p values written in bold are statistically significant (p < 0.05)

*p < 0.05 = significant difference

### Clinical problems

As presented in the first clinical case (Fig. 1), most of the participants (GDPs: 36.77% vs. PDs: 46.43%) suggested placing a GIC restoration (Table 6). However, the multivariate regression model demonstrated that composite resin was significantly the most relevant restorative material among participants who have more than 15 years of experience. For the second clinical case (Fig. 2), the majority of the participants supported the removal of all the affected MIH tissue until a healthy margin is reached. Moreover, composite resin was the material of choice for most GDPs (23.96%), while 35.71% of PDs preferred GIC restoration (Table 6). Moreover, the same treatment approach was preferred by dentists with more than 15 years of practice. However, dentists who encountered MIH patients on a weekly basis selected GIC restoration as the material of choice.

**Table 6:** Clinical problems

Question	Total, n (%)	GPDs, n (%)	PDs, n (%)
25. Which treatment would you prefer for a semi-erupted permanent molar with moderate MIH, post-eruptive fracture and sensitivity in the tooth in a seven-year-old patient?	471 (100)	359 (100)	112 (100)
Fluoride varnish	111 (23.57)	85 (23.68)	26 (23.21)
Glass ionomer cement	184 (39.07)	132 (36.77)	52 (46.43)
Composite	108 (22.93)	85 (23.68)	23 (20.54)
Extraction	6 (1.27)	3 (0.84)	3 (2.68)
Not sure	62 (13.16)	54 (15.04)	8 (7.14)
26. Which treatment option do you consider for a molar with delimited brown opacity without post-eruptive enamel fracture?	471 (100)	359 (100)	112 (100)
Eliminate all tissue affected by MIH until the healthy margin is reached and restore with:			
Composite restoration	104 (22.08)	86 (23.96)	18 (16.07)
Glass Ionomer restoration	114 (24.20)	74 (20.61)	40 (35.71)
Temporary restoration	14 (2.97)	13 (3.92)	1 (0.89)
Fluoride varnish	10 (2.12)	7 (1.95)	3 (2.68)
Sealant	12 (2.55)	6 (1.67)	6 (5.36)
Eliminate only the most affected tissue and restore with:			
Composite restoration	27 (5.73)	21 (5.85)	6 (5.36)
Glass Ionomer restoration	78 (16.56)	62 (17.27)	16 (14.29)
Temporary restoration	4 (0.85)	3 (0.84)	1 (0.89)
Fluoride varnish	4 (0.85)	3 (0.84)	1 (0.89)
Sealant	9 (1.91)	9 (2.51)	0 (0.00)
Do not eliminate any dental tissue and restore with:			
Composite restoration	11 (2.34)	9 (2.51)	2 (1.79)
Glass Ionomer restoration	14 (2.97)	10 (2.79)	4 (3.57)
Temporary restoration	5 (1.06)	5 (1.39)	0 (0.00)
Fluoride varnish	36 (7.64)	30 (8.36)	6 (5.36)
Sealant	29 (6.16)	21 (5.85)	8 (7.14)

GDPs, general dental practitioners; PDs, pediatric dentists

## Discussion

With MIH being a globally alarming problem and developing countries need to deal with the majority of MIH burden [15], there is a great need to address any knowledge gaps in such countries. To the best of the authors’ knowledge, this is the first questionnaire to investigate the knowledge, perceptions, attitudes, and clinical experiences on MIH among Syrian GDPs and PDs. We used an online questionnaire because it is more accurate, easier to use by participants, and increases the obtained response rate. The overall response rate was 36.31%. This survey has already been used in previous studies [10–13], which adds to its validity.

The results of this survey showed that PDs are more familiar with MIH, and could diagnose it better than GDPs. This could be due to the Syrian pediatric postgraduate program, which equips PDs with further knowledge concerning MIH [15]. A further explanation for this result is that 62.50% of PDs were still receiving information on MIH, with continuous education being the main source of knowledge. This result is consistent with the findings reported in Spain [10]. Most participants felt amelogenesis imperfecta was difficult to distinguish from MIH, whereas most GDPs reported that enamel hypoplasia was difficult to differentiate. A possible explanation for this finding is that amelogenesis imperfecta has diverse clinical features based on the enamel formation stage (hypoplastic, hypomature, or hypomineralized) [16]. Enamel hypoplasia is a quantitative enamel defect that could be difficult to distinguish from MIH-affected molars with post-eruptive breakdown [17]. This result agrees with previous findings reported in the UK [12]. In the present questionnaire, most respondents reported that genetic factors had a significant role in the etiology of MIH [18], with different views among GDPs and PDs. Similar results were reported in Saudi Arabia, Egypt, and Norway [19–21]. PDs have encountered more MIH-affected children during their practice and perceived that MIH prevalence is increasing in Syria. This could be due to PDs’ higher exposure to MIH-affected patients. Similar findings were reported in Spain, Egypt, and Hong Kong [10, 20, 22].

As expected, most GDPs were concerned about the negative effect of dental anxiety and poor appearance on children’s quality of life. A possible explanation for this finding is that behavior management is an essential part of any successful pediatric practice. Hence, PDs could be more skillful than GDPs and less likely to perceive anxiety as a problematic issue. However, children’s anxiety can be a limiting factor for children's behavior, even for PDs. In addition, Jalevik et al. [23] found that MIH-affected children had reported more dental fear and anxiety than their healthy counterparts. However, PDs were more concerned about pain experienced and missing school, which could be due to the fact that cases with severe MIH are referred to a PD. Therefore, it would require multiple visits and an advanced treatment approach. According to Fayle et al., adequate pain control could not be achieved in spite of injecting high doses of local anesthesia several times [7, 24].

Regarding restorative materials, stainless-steel crowns were the material of choice for molars with post-eruptive fractures, this result is not surprising as durability was the most decisive factor in the choice of dental materials for most participants. A recent study showed that the survival of stainless-steel crowns for MIH-affected molars was 94.4% after 24 months [25]. The same treatment option was used by dentists in the Australian survey [26]. Nevertheless, the majority of GDPs chose RMGIC as a suitable material for MIH-affected molars. Composite resin was the material of choice for both molars and incisors with opacities, it was recommended by Elhennawy et al. as well [27]. In addition, RMGIC was the least preferable restorative material among dentists with more than 15 years of practice for incisors and molars with opacities. The possible explanation for this finding is that RMGIC only serves as an interim restoration [28]. This could have led the majority of them to choose composite resin restoration because it is considered an optimal material for restoring all MIH severities [29]. Flowable composite resin was the most relevant restorative material for molars with post-eruptive eruption among dentists who encounter MIH patients on a weekly basis. This could be explained by the fact that flowable composite resin is used to cover MIH severe defects without cavity preparation in less cooperative pediatric patients [30]. Most PDs were most frequently encountered by yellow–brown demarcated opacities as a manifestation of MIH, consistent with findings in Spain, Hong Kong, and Portugal [10, 22, 31]. Most GDPs would refer MIH-affected children to a dental specialist for consultation and treatment, this reflects the insufficient training regarding MIH management. This explains the overwhelming majority of GDPs requesting further training concerning MIH treatment. These results are in agreement with similar findings in Egypt [20]. However, almost one-third of PDs would refer MIH-affected children as well. This could be due to the fact that in the present questionnaire, most PDs had fewer than 5 years of practice. This could have led them to refer severe cases to more experienced and older specialists.

For clinical case 1 (Fig. 1), GIC was the material of choice for an erupting molar with post-eruptive fracture, this was in agreement with Spanish dentists [10]. For clinical case 2 (Fig. 2), most respondents preferred the most invasive treatment by removing all the affected tissue until the healthy margin is reached. A possible explanation for this finding is that poor oral hygiene is a major burden in low-income countries [32]. As a consequence, dentists might embrace GV Black’s concept “extension for prevention”. In addition, PDs preferred to restore with an adhesive material such as composite resin, while GDPs preferred to restore with GIC as an interim restoration.

A good response rate is a strength of this study. However, it has drawbacks. Firstly, in questionnaires, most participants would select answers that they deem correct, rather than those truly reflect their practices and beliefs. Secondly, it was only conducted in the capital of Syria, Damascus. Thirdly, there is a dearth of official epidemiological data regarding MIH prevalence in Syria. Lastly, regarding the demographic profile, most participants were below the age of 30 and had fewer than 5 years of practice. Hence, the results of this questionnaire should be generalized with caution.

## Conclusions

Based on our findings, PDs were equipped with further knowledge regarding MIH. GDPs requested further training concerning MIH clinical management. There is a need for additional training and education for GDPs. Most participants perceived that the MIH prevalence is increasing in recent years, while there is a lack of data regarding MIH prevalence in Syria. The majority of GDPs did not receive any information on MIH, while PDs did with continuing education being the main source of knowledge. The materials of choice for restoring teeth with MIH were stainless steel crowns and composite resin.

## Data Availability

The datasets generated during and/or analyzed during the current study are available from the corresponding author on reasonable request.

## Abbreviations

MIH: Hypomineralized second primary molar
GDPs: Hypomineralized second primary molar
PDs: Hypomineralized second primary molar
PEB: Hypomineralized second primary molar
HSPM: Hypomineralized second primary molar
UK: United Kingdom
OHRQoL: Oral health-related quality of life
GIC: Glass ionomer cement
RMGIC: Resin modified glass ionomer cement
